# Light Harvesting in Fluctuating Environments: Evolution and Function of Antenna Proteins across Photosynthetic Lineage

**DOI:** 10.3390/plants10061184

**Published:** 2021-06-10

**Authors:** Pushan Bag

**Affiliations:** Department of Plant Physiology, Umeå Plant Science Centre, UPSC, Umeå University, 90736 Umeå, Sweden; pushan.bag@umu.se

**Keywords:** cyanobacteria, angiosperms, gymnosperms, light-harvesting complexes, phycobilisomes, photosynthesis, photoprotection, evolution

## Abstract

Photosynthesis is the major natural process that can harvest and harness solar energy into chemical energy. Photosynthesis is performed by a vast number of organisms from single cellular bacteria to higher plants and to make the process efficient, all photosynthetic organisms possess a special type of pigment protein complex(es) that is (are) capable of trapping light energy, known as photosynthetic light-harvesting antennae. From an evolutionary point of view, simpler (unicellular) organisms typically have a simple antenna, whereas higher plants possess complex antenna systems. The higher complexity of the antenna systems provides efficient fine tuning of photosynthesis. This relationship between the complexity of the antenna and the increasing complexity of the organism is mainly related to the remarkable acclimation capability of complex organisms under fluctuating environmental conditions. These antenna complexes not only harvest light, but also provide photoprotection under fluctuating light conditions. In this review, the evolution, structure, and function of different antenna complexes, from single cellular organisms to higher plants, are discussed in the context of the ability to acclimate and adapt to cope under fluctuating environmental conditions.

## 1. Introduction

Oxygenic photosynthesis is a natural process capable of harvesting solar energy into chemical energy and it is performed by green organisms, from cyanobacteria to higher plants. Oxygenic photosynthesis occurs in two phases; first, light energy is harvested and in the second phase, it is converted to chemical energy (glucose) [1]. Light harvesting takes place in the inner chloroplast membranes (thylakoids) by multi-unit pigment-protein complexes known as photosystems. The photosystems consist of two parts, the core and the antenna. The antenna proteins account for the harvesting of light energy and its transfer to the core. A charge separation takes place in the reaction centre of the core with the help of chlorophyll molecules. The nuclear encoded antenna proteins (sometimes known as the light-harvesting complex, Lhc) [2] surround the peripheral region of the core in order to maximise the energy capture [3,4]. Given the significance of the light harvesting process, the light-harvesting antennae have been the major focus of photosynthesis research since the 1980s [5] and continue to be so to date. Based on the quality and quantity of the available light in the niche of the organisms, the antennae were found to be very diverse across the lineage of green organisms [2,6]. The first important landmark in the research of light-harvesting antenna proteins came with the development of DNA sequencing in the late 1970s. Afterwards, the complete genome sequence of *Arabidopsis thaliana* became available in 2000 [7]. *Arabidopsis* became the “model organism” for plant research and also for the characterization of the antenna proteins in higher plants, although the antenna systems in other species such as the tomato or the Scots pine were studied in the 1980s and 1990s [8]. Later, due to advanced genome sequencing techniques, the *Arabidopsis* homologues of the antenna proteins were identified in several other species. To date, photosynthetic antenna related genes have been identified in ten different clades [9] of organisms, including phycobilisomes in ancient cyanobacterial species to Lhcs in higher plants.

Phycobilisomes, the major light-harvesting antenna system in cyanobacteria, is not a member of the Lhc super family but has a special significance as it is the most primitive form of the complex multimeric antenna systems [10,11,12,13,14,15]. Apart from phycobilisomes, there are huge numbers of Lhc-like proteins, such as FCPs (Fucoxanthin chlorophyll a/b binding proteins) [16,17,18,19,20,21] etc., that span across the photosynthetic lineage. The Lhc super family proteins are either named based on their association with photosystems or their function. LhcA and LhcB are associated with photosystem I and photosystem II, respectively [2,8], whereas LhcX and ELIP (early light-induced protein) are named by functionality. Lhcs that are named based on their association with photosystems are mostly “true Lhcs”, as their major function is light harvesting, whereas other Lhcs, known as Lhc-like proteins, are mainly involved in energy dissipation and stress responses rather than light harvesting. Although light harvesting is not their major function, Lhc-like proteins were named so due to the presence of a hydrophobic “generic Lhc motif” of approximately 22 aa long. Even with this functional diversity, most of the Lhc super family proteins have similar structures; three membrane-spanning helices, of which helices one and three are homologous, bind chlorophylls and carotenoids [2]. This functional and structural similarity or diversity make the Lhcs the most conspicuous proteins in green organisms. This has motivated scientists to discover new Lhcs and study the known ones with new and emerging techniques repeatedly, which has enormously improved our understanding of the overall photosynthetic light harvesting process.

In 1999, a perspective article on Lhc genes was published and described the Lhc family gene [2]. Over the following ten years, several reviews have compiled evidence of the roles of different Lhc proteins [22,23,24]. In the last decade, several Lhc proteins have been identified and their possible roles have also been elucidated in several additional species [6,25,26,27]. In this review, an updated comprehensive summary of the evolution, structural and functional properties of light-harvesting antenna proteins are discussed in the context of their possible roles in plant acclimation and adaptation to fluctuating environmental conditions and how the diversity in light-harvesting antennae is linked to the evolution of photosynthetic organisms.

## 2. Evolutionary Aspects of Light-Harvesting Antennae

**Figure 1:**
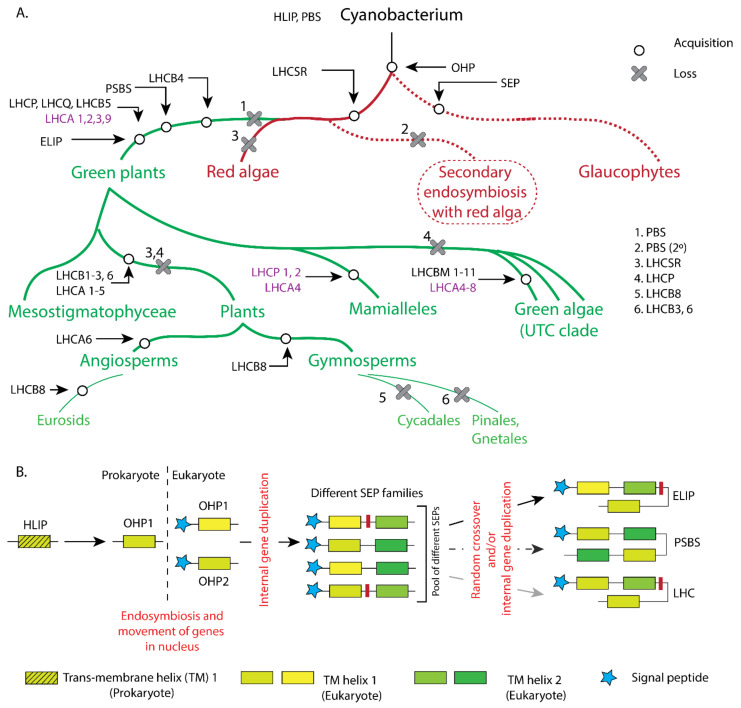
The evolution of the light-harvesting antenna proteins across the photosynthetic lineage from cyanobacteria to higher plants. (**A**) The loss and gain of the different light harvesting proteins and their homologues during the evolution of the photosynthetic lineage. Proteins (in magenta) share a similar genetic background, but are slightly different from each other, such as algae type LhcAs (in magenta) that are slightly different from the plant type LhcAs (in black). (**B**) Genetic events that may have occurred during evolution to give rise to higher plant antennae from their prokaryotic ancestors. The evolution of ELIP, PsbS and Lhc from different SEP families occurred via independent events.

The origin of life from one common ancestor, as described by the father of evolution, Charles Darwin, in his book *On the Origin of Species* [28], is the most widely accepted foundational concept of biology, that leads to the proposition of natural selection theory as a driving force of evolution. Although the scientific discussion on the relationship between natural selection and evolution remains out of the scope of this article, the natural behaviour of plants to grow better in their natural habitat has, no doubt, very strong ties with their evolution. As Darwin noted in his book, *The Movements and Habits of Climbing Plants* [29], plants tend to move towards a light source, which is a classic example of how the growth environment influences the acclimation (short term response), the adaptation and the evolution of plants in different habitats [30]. This not only applies to the higher plants, but also explains the long evolutionary history of all photosynthetic organisms acquiring different photosynthetic pigments in order to thrive under various environments [23,30,31]. As photosynthetic organisms evolved from single cellular prokaryotic bacteria [32] to multicellular eukaryotic land plants, the need for diverse light harvesting apparatus increased concomitantly. In the following sections, the structural and functional characterization of light harvesting apparatus from cyanobacteria to higher plants are compiled through the evolution of the photosynthetic lineage (Figure 1).

### 2.1. Light-Harvesting Antennae in Cyanobacteria 

**Figure 2:**
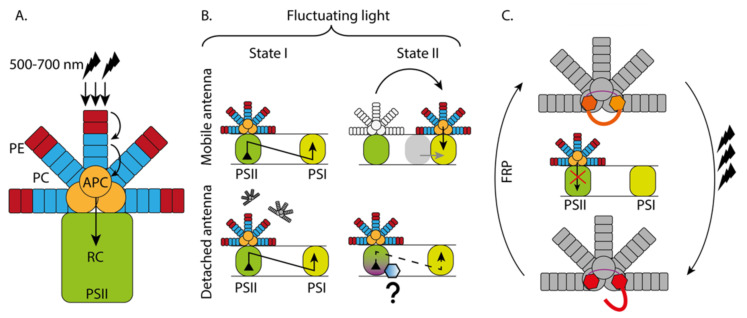
The functional properties of the cyanobacterial phycobilisome antenna (PBS) and orange carotenoid proteins (OCP) under fluctuating light conditions. (**A**) Structure of the PBS and the energy flow in the antenna system (**B**) Mobile and detached antenna models proposed for the acclimation of the light harvesting systems under fluctuating light regimes in the cyanobacteria (state transition). In the mobile antenna model, in state II, energy spillover is enabled by the physical contact of PSII with PSI. In the detached antenna model, in state II, the PBS remains with PSII that is quenched by a yet unidentified mechanism. (**C**) Excitation energy quenching by orange carotenoid proteins (OCP) in PSB. In high light, the conformation of OCP changes and excess energy is dissipated from the PBS. After the high light condition has passed, FRPs (fluorescence recovery proteins) bind to the red conformation of OCP, OCP dissociates from the PBS and the NPQ mechanism is turned off. ACP = allophycocyanin, PE = phycoerythrin, PC = phycocyanin, RC = reaction centre.

Based on current geological understanding, the Earth was formed 4.5 billion years (Ga) ago by accretion from solar nebula and life on Earth appeared in its primitive form around 4 Ga [33] ago and continued evolving into the most ancient photosynthetic organisms around 3.5–3.3 Ga [34] ago. The last known common ancestors of higher plants, the cyanobacteria, appeared much later, around 2.5 Ga ago and are considered to be the origin of oxygenic photosynthesis [35]. The current variants of the photosynthetic cyanobacterial species depend on the family of the phycobiliprotein (PB) complexes known as phycobilisomes (PBSs) for harvesting light [36]. PBSs are the only form of light-harvesting antenna that evolved from globin proteins [14] containing one type of chromophore covalently bound to PBS [37]. Structurally, PBSs assemble with the help of linker peptides [13]. The assembly of PBS makes the overall antenna complex very large and capable of binding on top of the photosystems to perform light harvesting. Cyanobacteria lack “true LCHs”, but it has HLIP (high light inducible protein) proteins that have similar first or third helix structures to those of Lhcs, hence HLIPs belong to the Lhc super family. HLIPs were discovered in cyanobacteria under high light adaption [38]. There are four main HLIP proteins named from A to D and all of them bind to PSII [39]. HLIP C and D are reported to bind in the early PSII assembly and HLIP A and B bind in the later assembly phase, but also, they can bind to the complete PSII core. HLIPs are present in cyanobacteria and the plastid genomes of red algae, glaucophytes and the cryptophytes [40]. In addition to PBS and HLIP proteins, cyanobacteria also contain a water-soluble photosensitive protein, Orange Carotenoid Protein (OCP), that can account for photoprotection that can efficiently quench light energy (Figure 2) [41].

### 2.2. Light-Harvesting Antenna in Red Lineages

**Figure 3:**
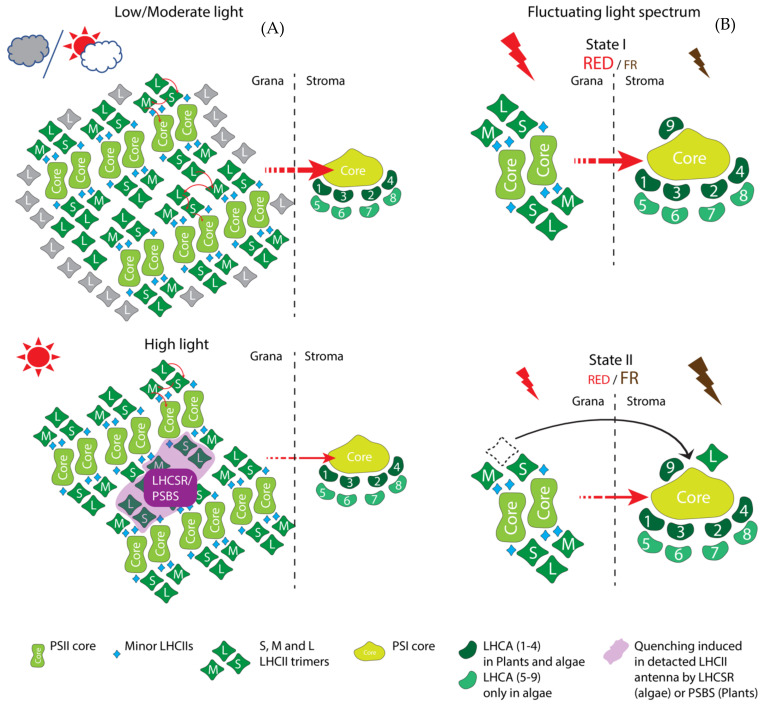
Functional insight into green algae and the plant antenna (Lhc) and the photoprotection proteins. (**A**) In low or moderate light, maximum energy from sunlight is harvested and channelled to PSI for downstream mechanisms photosynthesis. Loosely bound LCHII L trimers (in grey) associate with PSII and increase the antenna size in low light conditions or in shaded leaves. (**B**). In high light conditions, the LhcII antenna system is quenched by either the LhcSR (in algae) or the PsbS (in higher plants). This prevents the over-excitation of the PSI as less energy is harvested by PSII. (**C**)The shift from red to far red-light causes L trimers from PSII–LhcII complexes to move to PSI where they serve to increase the PSI antenna size as far-red light preferentially excites PSI.

Later in evolution, plastid acquisition took place via endosymbiosis and gave rise to red lineages of eukaryotic organisms. Eukaryotic red lineage organisms have PBS and HLIPs or HLIP-like homologues known as LILs (Light-harvesting-like) family proteins. Theses LILs contain one transit peptide as a signature of a eukaryotic organism (nuclear encoded) and either one, two or three transmembrane chlorophyll-binding domains. The number of chlorophyll-binding domains defines their nomenclature as OHP (one-helix protein), SEP (stress-enhanced proteins) or ELIP (early light-inducible proteins, only in green lineages). Within the red lineages, unicellular Glaucophytes contain an OHP and SEP. Compared to the glaucophytes, more evolved red algae and diatoms acquired Lhc-like proteins currently known as LhcSR (Light-Harvesting Complex Stress-Related proteins, previously known as LI818 [42] in green algae). LhcSR proteins [43] are mostly responsible for photoprotection (energy dependent quenching, qE) [42]. The evolution of LhcSR proteins is widely believed to have occurred due to internal gene duplication from different SEPs [44]. Later, when the red lineages diverged from the green lineages, these LhcSR homologues were also carried over into the green lineages and are thus found in *Chlamydomonas* and other genera [43]. Although the antenna systems are divergent between the green and red lineages, indicating that they have evolved separately, the shared presence of LhcSR clade proteins proves that they are the first known common ancestral eukaryotic Lhcs [45]. In the red algae, in addition to the LhcSR, there are three more groups of Lhc or Lhc-like proteins, namely LhcR (red algae origin ‘Rhodophyta’), LhcF (Fucoxanthin-Chl a/c binding proteins) and LhcZ (the function was unknown at that time [43]). All the Lhcs in the red algae are functionally limited to PSI and based on their binding motifs, they have the capability to bind to carotenoids as well. Due to the lack of evidence, the evolution of the Lhcs in the red lineages cannot be determined explicitly. However, the common ancestry of the LSR protein certainly makes it one of the major candidates from which LhcR and LhcF may have evolved (Figure 3A,B).

### 2.3. Light-Harvesting Antennae in Green Algae

The rise of green algae lineages (Chlorophyta) [46] from the red algae lineages was accompanied by a loss of phycobilisomes (PBS, red antenna). The protein family of chlorophyll a/b binding Lhcs that had already emerged in the red algae was further expanded and diverged in the green lineages, giving rise to separate PSII and PSI antenna systems [47]. As per current understanding, the earliest Lhcs attached to PSII were LhcB4 (CP29) and LhcB5 (CP26) minor antennae. As all early green algae lineages (Viridiphytae) contain LhcB4 (or LhcB4-like proteins), it is safe to infer that LhcB4 was the first among these two antennae [48]. After LhcB4 and LhcB5, green algae evolved several other PSII and PSI antenna systems, namely, LhcQ, LhcP (Major antenna—Lhc Prasinophyte), LhcA2, LhcA3 and LhcA9. All these Lhcs evolved by internal gene duplication or by some unequal cross-over of tandem SEPs of one class [44]. Among these proteins, LhcP is the most conspicuous one, due to its ability to bind Chl a, b, c and several carotenoids [49]. As with the aforementioned Lhcs, a similar duplication event between different SEP classes gave rise to a four-helix protein, now named as PsbS [50] (S subunit of PSII). In addition, another three-domain proteins evolved, most likely evolved from pool of SEPs but independent of PsbS or the ancestry of Lhcs, known as ELIPs [44]. Further down the evolutionary tree, LhcPs were lost in the UTC clade that separated from Mamiellaceae [23]. Rather, LhcAs and LhcBs became more divergent in the UTC clade and evolved into additional PSII antenna proteins LhcBM 1-11 [23] and PSI antenna proteins LhcA 4–8 [43]. In the Mamiellaceae clade only one PSI Lhc, i.e., LhcA4 and two homologues of LhcP, i.e., LhcP1 and LhcP2 [43] emerged (Figure 3A,B). 

### 2.4. Light Harvesting in Terrestrial Green Lineage

As per current understanding, the clade Chlorophyta [36] contains mainly (~90%) freshwater organisms [51]. The more evolved Streptophyta clade contains terrestrial organisms from which land plants evolved later [52]. During the evolution of terrestrial green organisms from freshwater algae, PSII Lhcs were acquired, i.e., the LhcB9 minor antenna [23] and LhcB3 major antenna appeared. Furthermore, when higher plants evolved, LhcII became more diverse, and LhcB1 and LhcB2 emerged among the major antennae, whereas LhcB7 and LhcB8 emerged among the minor antennae [43]. At the same time, all LhcBMs were lost along with LhcB9. The evolution of the PSI antennae is rather more interesting: LhcA4 and LhcA5 appeared and LhcA9 was lost. Moreover, LhcA2 which was already present in the green algae, evolved to another homologue, LhcA2 type II and hence the LhcA2 in Chlorophyta is named as LhcA2 type I. Along with Lhcs, PsbS remained as a main photoprotection protein, whereas the other homologues of LI818/LhcSR were lost. Finally, further down the evolutionary tree, when higher plants evolved and Gymnosperms and Angiosperms diverged, some lineages of the gymnosperms lost LhcB3 and LhcB6 from PSII and LhcA5 from the PSI antenna cluster (Pinales and Gentales order). Interestingly, some gymnosperms also evolved different isoforms of LhcB1 (PSII Lhc), namely LhcB1_A and LhcB1_B [2,26] (see next sections). In the case of angiosperms, LhcA6 appeared in the PSI antenna complexes and most of them did not retain LhcB8 in the PSII antenna system.

In summary, it becomes clear that the most obvious reason behind the evolution of the highly advanced antennae from the ancient ones, i.e., from phycobilisomes to Lhcs in higher plants, has been due to chance and random genetic events such as chromosome cross over and gene duplication etc., that have introduced the variation in Lhcs observed across the green lineages. Moreover, environmental niches have played a significant role in this diversification of the antenna systems to a great extent, enabling prosperous light harvesting and the survival of photosynthetic organisms (Figure 3A,B).

## 3. The Functional Properties of Light-Harvesting Antennae in Relation to Coping Mechanisms under Fluctuating Environmental Conditions

As mentioned above, the light-harvesting antenna systems diversified during the course of evolution in order to provide short-term acclimation and long-term adaptation to cope with fluctuating environmental conditions. While single cellular prokaryotic organisms can deal with short-term fluctuations with their limited antenna systems, they fail to cope with prolonged stress. On the other hand, higher eukaryotes are well equipped with their decorated antenna systems to deal with both short-term and long-term changes in their growth environment. In the following sections, the functional roles of the antenna proteins in different photosynthetic organisms are summarised in order of their appearance in the evolutionary tree. Furthermore, the possible relationship between the complexity and function of the antenna proteins and the organism’s habitat is discussed.

### 3.1. Phycobilisome Antenna System (PBS)

#### 3.1.1. Light Harvesting Capacity of PBS

Th phycobilisome (PBS) is a large umbrella-like structure that can absorb light from the blue-green to the red region of the visible light spectrum (500–700 nm) [53]. This feature is very unique for cyanobacteria, red algae and glaucophytes since other green organisms (green algae, higher plants) absorb light mainly in the blue (430–480nm) and red (680–700 nm) regions and not in the green, yellow and orange regions [54]. The uniqueness of the PBS light absorption capacity is enabled by the pigments known as phycobilins. In general, the PBS comprises of three types of phycobilins: phycoerythrin (PE), phycocyanin (PC) that forms the outer rods and allophycocyanin (APC) that forms the phycobilisome core [11,55,56]. This large antenna-like structure is anchored to the thylakoid membrane with the help of a linker peptide (Lcm). Although the PBS composition varies based on the organism, in general, the absorbed energy is funnelled to a lower energy state (PE_575nm_ > PC_640nm_ > APC_660nm_ > Lcm_680nm_ > PSII RC) and transferred to the PSII reaction centre which absorbs around 680 nm [47]. The energy transfer process (energy trapping followed by relaxation) from different phycobilins in the PBS to the reaction centre of PSII happens in picoseconds (ps). Based on the composition of the PBS and the growth conditions of the organism, these energy transfer times may vary, but in general, the trapping times in the rods are around 10 ps, whereas in the phycobilisome core (APCs) it varies from fs (femtoseconds) [57] to tens of ps [56] (Figure 2A).

#### 3.1.2. PBS Functioning under Different Light Conditions 

How the PBS functions under different light conditions is under debate and therefore there are several models and hypotheses that try to explain how the PBS behaves in response to changing incident light. This debate arises from the conspicuous feature of the PBS to bind both PSII and PSI under different light regimes. There are two major hypotheses: the PBS mobile model and the PBS detachment model [58]. In the first model, the PBS remains bound to PSII under red light (State I), since red light preferentially excites PSII rather than PSI and triggers the downstream process of charge separation in the reaction centre [59]. When state II is triggered under far-red light, the PBS migrates to PSI and shifts the excitation balance towards PSI. Moreover, it has also been proposed that in state II, PSII and PSI form a physically contacted complex and the PBS serves as an antenna for balancing the excitation between the two photosystems, as energy spillover from PSII to PSI takes place [10,60,61]. This model remains highly debated [58]. In the second model, in state I, the PBS is believed to be in a detached form and for this reason has a prolonged emission compared to state II, where it remains bound to PSII. An improved version of the second model proposes that in state I, PSII remains active as in the first model and the PBS is partially detached. In state II, the PBS remains quenched but attached to PSII (by an unknown mechanism) allowing a partial energy transfer (Figure 2B). This state transition mechanism in cyanobacteria via phycobilisomes differs significantly from the more widely known ‘state transition’ phenomena in higher plants and algae [62,63,64,65,66].

### 3.2. Orange Carotenoid Proteins (OCPs) Facilitate Short-Term Acclimation to High Light

Under high levels of irradiance, the cyanobacterial peripheral antenna transfers a large amount of energy downstream that causes damage to the photosystem reaction centres. Cyanobacteria respond to long-term high light exposure by increasing the abundance of chlorophylls that are preferentially channelled to trimeric PSI. This mechanism enables the survival of the organism at 300 μmol m^−2^ s^−1^ of irradiance [67], but further increases in the irradiance cause permanent damage to the photosystems. 

In cyanobacteria, the mechanism to respond to high irradiance is very simple and the excess energy is mainly quenched by orange carotenoid proteins (OCPs) [68]. OCPs are composed of one α-helical N-terminal domain (NTD), one α/β mixed C-terminal domain (CTD), a linker peptide and a keto-carotenoid that spans across both NTD and CTD [37,41,68,69,70]. OCPs can bind to several carotenoids and perform thermal energy dissipation (Non photochemical—NPQ) [71,72]. Upon high light exposure, OCP carotenoids change conformation and translocate in NTD, which makes the OCP change to the active red form, which is efficient in dissipating thermal energy [73]. Upon the conversion from orange to red conformation, OCP interacts with the APC of the PBS and dissipates energy from the PBS and thus prevents excess energy passing to the reaction centre. When the high irradiance conditions have passed, the FRPs (fluorescence recovery proteins) bind to the red conformation of OCP that facilitates the dissociation of OCP from the PBS, switching the NPQ mechanism off [74]. Apart from NPQ, OCPs are also capable of alleviating oxidative stress [71] and other stress conditions [75]. It is obvious that OCPs have numerous functions that help these primitive organisms to cope under stressful environmental conditions (Figure 2C).

### 3.3. HLIP and Eukaryotic LIL Proteins

#### 3.3.1. The HLIP Family Is Associated with High Light and Oxidative Stress Responses

HLIPs are a group of proteins that play photoprotective roles in both cyanobacteria and red algae, whereas the importance of HLIPs in photoprotection in higher plants remains elusive [39]. In cyanobacteria, HLIPs protect PSII under high irradiance [38] by transferring energy from the excited chlorophylls to carotenoids [76]. Reports suggest that HLIPs are efficient in energy quenching in free chlorophylls that otherwise can be lethal for the cell due to photodynamic characteristics. HLIPs also provide protection from other stress conditions [77]. Another function of HLIPs is related to their role during PSII assembly. HLIPs can bind and protect the pre-assembly and mid-assembly of PSII complexes in cyanobacteria [39]. HLIPs are also known to take part in chlorophyll metabolism and chlorophyll recycling [39,78]. Considering the overlap between chlorophyll metabolism and the biosynthesis of chlorophyll containing proteins, it is obvious that HLIPs play major roles in the formation of the photosynthetic pigment protein complexes. Furthermore, HLIPs were proposed to have a role in maintaining PSI stability [79], but such reports remain scarce. Nevertheless, the aforementioned roles of HLIPs are regarded as less important than that of OCPs, since many of these functions can be performed without HLIPs at a slower pace. Overall, HLIPs across the green lineages can be considered as auxiliary but not as essential proteins.

#### 3.3.2. OHP Family

Among the eukaryotic lineage, OHP1 and OHP2 function in red and green algae and land plants. OHP1 and OHP2 are known to accumulate in high light conditions [2] and they are associated with PSI [80]. Both OHPs play a crucial role in the assembly of PSII and PSI [81]. Moreover, *OHP1* mutants suffer from constant oxidative stress, which confirms their function in maintaining redox homeostasis in the chloroplast [81]. In a recent study, OHPs were further suggested to bind to chlorophyll and carotenoid molecules and thus prevent the pigments from interacting with oxygen [82]. 

#### 3.3.3. SEP Family

Stress enhanced proteins (SEPs) are two helix proteins that are strictly eukaryotic in origin and present in red and green algae and land plants [2,81]. The function of SEPs is related to stress responses, either light stress or oxidative stress [80]. The detailed functions of SEP1 and 2 (LIL1 and 2) are very poorly reported, whereas reports about SEP3 (LIL3) suggest that it plays a role in chlorophyll biosynthesis. In barley, it appears to participate in the delivery of protochlorophyllide a to Chl synthase and the transfer of esterified Chl from the enzyme to the chlorophyll binding proteins [80]. In *Arabidopsis*, LIL3 plays a role in the biosynthesis of tocopherols and chlorophylls by stabilizing geranylgeranyl reductase [83].

#### 3.3.4. ELIP Family

Early light inducible proteins (ELIPs) are three-helix proteins only present in the eukaryotic green lineages [80]. ELIPs in the green moss *Physcomitrella sp* provide protection against photo-oxidative stresses [84]. In higher plants, ELIPs accumulate during dark to light transition [85] and in high light stress conditions [86,87]. ELIPs are reported to be localized with the LhcII antenna systems in *Arabidopsis* [88] and respond to blue and UV light in an intensity dependent manner [86,89,90,91]. Since they respond to blue and UV light, ELIPs are correlated with PSII photoinhibition and photodamage of the reaction centre [87,88,89]. ELIPs are capable of harbouring Chl a and lutein [92], but they exhibit only weak excitonic coupling within Chl a molecules. Thus, their interaction with lutein suggests that they may play a prominent role in photoprotection rather than in light harvesting [92].

In short, these HLIP and LIL family proteins present in both pro- and eukaryotic organisms are involved in high light and oxidative stress responses or chlorophyll metabolism rather than light harvesting and hence they are not considered as ‘true Lhcs’ but as Lhc-like proteins.

### 3.4. LhcSR Family Proteins Act in Non-Photochemical Quenching

Light-Harvesting Complex Stress-Related (LhcSR) proteins are found in red (LI818) and green algae (LhcSR) but not in higher plants [23,93]. LhcSR proteins are widely known to regulate thermal energy dissipation (qE type of NPQ) [93,94,95,96]. Four main types of LhcSRs are known to date: LhcSR1, 2 and 3 in *Chlamydomonas* and mosses and LI818 type in red algae [23]. LhcSR1 and 3 are reported to perform energy-dependent quenching, but their quenching capability varies due to their different ability to occupy carotenoids in the quenching site (L2 site) in *Chlamydomonas reinhardtii* [94]. LhcSR1 and LhcSR2 are involved in NPQ in moss *Physcomitrella patens* [97]. In addition, in *Chlamydomonas*, the expression of *LhcSR1* is upregulated by high CO_2_ levels [97] and LhcSR1 is involved in the UV stress response [98,99,100]. Among the LhcSRs, the most important one appears to be LhcSR3. Although LhcSR3 is found with both PSI and PSII [101], the energy dissipation is mostly linked with PSII [93,94]. Only one report proposes that LhcSR3 is also responsible for energy dissipation in PSI [102]. LhcSR3 binds to C2S2 type PSII-LhcII supercomplexes in grana regions [103] and it is shown to be essential for the coupling and the de-coupling of LhcII from the PSII core [96]. LhcSR3 can also migrate between PSII and PSI during state transition [99] and interact with the PSI-LhcI-FNR (Ferredoxin-NADP(+) reductase) complex upon phosphorylation by STT7 kinase [63,66] (STATE TRANSITION DEFICIENT 7). Although the evidence related to the role of LhcSR3 in PSI remains scarce, it is hypothesized that in high light conditions, LhcSR3 most likely induces photoinhibition upon the downregulation of Pgrl1 (proton gradient regulator like 1) mediated cyclic electron flow and prevents the degradation of the iron sulphur cluster [66,101] (Figure 3). 

### 3.5. Lhc Family the ‘True Lhcs’ Are the Most Complex and Diverse Group of Light-Harvesting Antennae

Light-harvesting complex (Lhc) proteins are present from the red lineages to the most evolved higher plants. Lhcs first appeared as the minor antenna (LhcB4) and major antenna (LhcA and LhcQ) from LhcSR in red algae. In the following section, “true Lhcs” are presented based on their appearance from lower clade organisms to higher plants and based on their association with the photosystems.

#### 3.5.1. Minor PSII Antenna—LhcB4 to LhcB9

The first Lhc antenna that appeared in green eukaryotes was the LhcB4 minor antenna [43]. Later, LhcB5-9 emerged along the course of evolution as discussed previously. It is widely accepted that the minor antennae take part in light harvesting and the dissipation of excess energy by NPQ [2,50,104]. Minor antennae, LhcB4-6, are known to form quenching sites for PsbS [105] and the deletion of encoding genes results in the slow induction of NPQ [106], although the maximal amplitude of NPQ in the mutant plants remains similar to the wild type [105]. This indicated that thermal dissipation was not the main function of these Lhcs. Instead, several recent reports suggest that minor antennae systems promote the assembly and stability of PSII–LhcII supercomplexes in the membrane and help to maintain the structural organization of the photosystems under fluctuating light conditions [107,108,109]. Evidence suggests that LhcB5 and LhcB6 act as a bridge between the PSII core and the major LhcIIs and thus allow flexible plastoquinone (PQ) diffusion and efficient linear electron flow [110]. On the other hand, recent findings suggest that the peculiar accumulation of LhcB4 in plants acclimated to moderate and high light conditions [108,109] is explained by the function of LhcB4 in preventing the photoinhibition of PSII [111]. This clears the confusion surrounding its role in NPQ: the minor antennae do not affect the fast NPQ components but helps in the formation of the slow NPQ components, which is essential for preventing photoinhibition. Apart from light harvesting and NPQ, minor antennae also protect PSII from oxidative damage [100]. LhcB9 is another PSII minor antenna only present in mosses and, in low light, it associates with the PSI antenna and forms a unique PSI megacomplex (apart from the normal one PSI-LhcI) [112] that is more like the aquatic green algae *Chlamydomonas* [102] (see next section) (Figure 3). Recent reports also suggest that the minor antenna CP24 can function to create a binding cleft for PsbS to PSII–LhcII supercomplexes [113].

#### 3.5.2. Major PSII Antenna—LhcP and LhcQ

LhcPs are prasinophyte-specific (aquatic green algae) proteins that bind chlorophyll a, b and c-like pigments and a number of unusual carotenoids [12]. LhcPs are mostly PSII antennae although they possess the capability to bind with both the PSI and the PSII reaction centres [49]. Apart from light harvesting, no other roles have been reported yet for LhcPs. However, it is not too ambitious to hypothesise that these antennae may play additional roles during the state transition in aquatic green algae in fluctuating light conditions.

LhcQs are typically found in green algae and higher plants but not in mosses [43,114]. The trimeric organization motif in LhcQs, as in other major LhcII proteins in higher plants [12], suggests that LhcQs are involved in light harvesting similar to other peripheral trimeric antenna systems.

#### 3.5.3. Major PSII Antennae—LhcBM1 to 11 

LhcBMs are specific to green algae and mosses but are not found in higher plants [114]. There are nine LhcBMs found in algae and 13 LhcBMs in mosses, where they serve as major PSII antennae [23]. LhcBM4, LhcBM6 and LhcBM8 are reported to be involved in light harvesting and to serve as potential NPQ sites [102]. LhcBM1 participates in the qE type of quenching by being the main interacting site for LhcSR3 [115,116], whereas LhcBM2, LhcBM5 and LhcBM7 are known to be involved in state transition [64,117]. Moreover, LhcBM5 can serve as a PSI antenna in low light conditions (state II) [118] and LhcBM9 is important for protecting PSII during nutrient starvation [119,120].

#### 3.5.4. Major PSII Antennae—LhcB1 to 3

In higher plants, LhcB1, LhcB2 and LhcB3 are the major antenna proteins that contain a WYGPDR motif that is necessary for the trimeric conformation of LhcII and can act as a site of the qE type of NPQ [23]. Among these antennae, LhcB1 is essential for the regulation of grana stacking (that is regulated by surface charge of PSII–LhcII super-complex moiety) [121], whereas LhcB2 is essential for the efficient state transition in response to the shift in the light spectrum from red to far-red [65,121]. In natural, shaded growth conditions, all three LhcBs accumulate to maximise light capture. These three major Lhcs form either homotrimers or heterotrimers and the trimeric organisation of LhcII is mostly characterized by their association with the PSII core as strongly bound (S), moderately bound (M) and loosely bound (L) LhcII trimers [122]. Whereas S and M trimers are associated to PSII, L trimers can migrate to PSI during state transition. Among these trimers, LhcB3 is only found in M trimers [123]. The role of LhcB3 is very conspicuous—LhcB3 affects the macromolecular structure of PSII supercomplexes and the rate of state transition without affecting the maximum extent of state transition. This suggests that the light harvesting role of LhcB3 under changing environmental conditions is confined to the structural stability of PSII complexes, LhcB1 provides structural stability to the macrodomains of thylakoids and LhcB2 is directly involved in state transition (Figure 3). 

Interestingly, gymnosperms lack LhcB3 and LhcB6 [124] but possess an additional isoform of LhcB1 named LhcB1_A, which displays a large extent of phosphorylation and a great potential to modulate the thylakoid ultrastructure during winter acclimation [26].

#### 3.5.5. PSI Antenna—LhcAs

LhcAs are major and minor PSI antennae found in green algae and higher plants. In higher plants, among the major four LhcAs, LhcA1/4 and LhcA2/3 form dimeric structures and bind on one side of PSI, keeping the other side open for the docking of LhcII trimers in state II conditions [125,126,127]. All four LhcAs are involved in light harvesting and their binding to PSI is very specific and cannot be substituted, with the exception of LhcA4 that can be complemented by LhcA5 to form a native PSI –LhcI complex [128]. Recently it has also been shown that LhcA2/3 can also bind to LhcII trimers and the complete LhcA1–4 antenna can facilitate a PSI supercomplex assembly with cytochrome b6f (Cytb6f) or a NDH (NAD[P]H dehydrogenase) complex [129]. LhcA5 and LhcA6 are reported to help in type II NDH binding and can facilitate an NDH-mediated cyclic electron flow [130].

In green algae, there are nine LhcAs reported to date and it is worth noting that there is no direct evolutionary relationship between the individual LhcAs in *Chlamydomonas* and higher plants. According to their spectra, LhcA1, LhcA3 and LhcA7 are regarded as blue Lhcs (spectrally blue shifted), LhcA5, LhcA6 and LhcA8 as intermediates and LhcA2, LhcA4 and LhcA9 as red Lhcs (spectrally red shifted) [125]. Interestingly, LhcA2 and LhcA9 are also reported to be replaced by Cytb6f to form a stable PSI–Cytb6f complex which triggers a cyclic electron flow under anaerobic conditions [62] (Figure 3).

### 3.6. Photosystem II Subunit S (PsbS) 

PsbS is a distant homologue of LI818 of the early eukaryotes confined to green algae and higher plants [42,45,131]. PsBs is solely responsible for the qE type of NPQ in higher plants that lack LhcSR. PsbS is known to interact with LhcIIs to induce quenching in the PSII antenna system [118,132,133,134], rather than directly binding to the reaction centre [119]. It has also been shown that PsbS protects PSI from high light stress and in fluctuating environments it interacts with carotenoids in a complex mechanism to dissipate excess energy [132,135,136,137]. In *Chlamydomonas*, PsbS was first found under UV light stress [100] and to date it is known to regulate qE together with LhcSR3 in high light conditions [138,139]. Moreover, in conifers, the phosphorylated isoform of the PsbS homologue has been reported to facilitate sustained winter quenching [26] together with direct energy transfer [27]. It has also been demonstrated that the overexpression of PsbS in tobacco increases water-use efficiency due to a reduced stomatal opening in response to light, resulting in promoted growth in field conditions [140] (Figure 3). This indicates that the photosystem antenna complexes can have multiple functions that affect the overall plant performance.

Apart from the above-mentioned antenna proteins, there remains a vast number of organisms that have hugely diverse antenna systems that have evolved under different environmental conditions, such as oxygenic *Prochlorococcus* which has evolved under low oxygenic conditions [141] or algae (*Chlorella ohadii*) which grows in dessert soil crusts under extreme illuminations [142,143] etc. 

## 4. Conclusions

**Figure 4:**
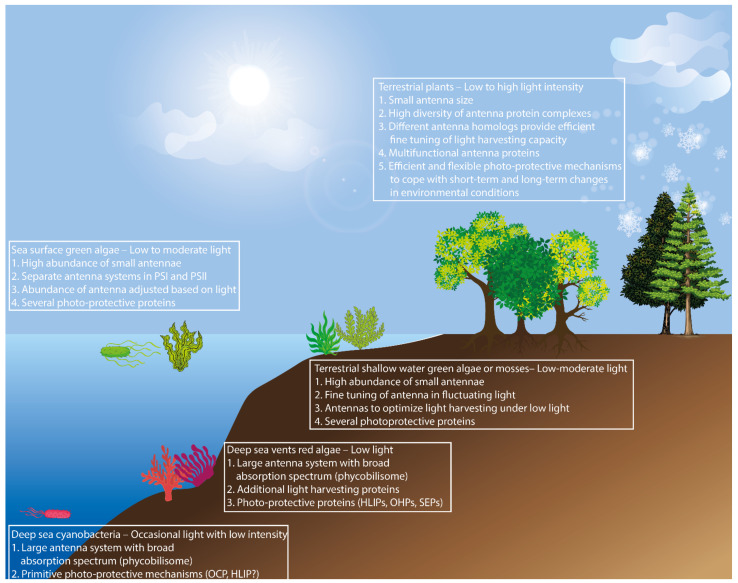
Visual illustration of possible correlation between the evolution and function of light-harvesting antennae and the light environment of the photosynthetic organism.

It is clear that the evolution of light-harvesting antennae has been shaped by a big element of chance and that the growth environment has played a very significant role as the driving force of the diversification of antenna systems from specific light absorbing proteins found in ancient photosynthetic bacteria, to complex and multifunctional antenna systems found in higher plants (Figure 4). 

When the first photosynthetic cyanobacteria appeared on the ocean floor, they had a huge umbrella-like antenna (PBS) with the capacity to absorb light across a wide light spectrum. This allowed them to harvest an ample amount of light in the deep-sea environment where light is scarce and is encountered only occasionally. Thus, the first photosynthetic organisms possessed only primitive OCPs that could handle short-term exposure to high light efficiently. Similarly, the first eukaryotic photosynthetic red algae that appeared in the deep-sea vents had similar large antenna systems (PBS) but, on the other hand, random genetic events gave rise to protective proteins and ancient forms of light-harvesting antenna proteins. Later, when green algae evolved and started to emerge on the ocean surface, the need for a large antenna system became obsolete due to higher irradiance. Most probably, these green algae lost their PBS antenna and instead their Lhcs evolved and diversified. As a result, several Lhcs binding to photosystems and some LhcIIs capable of binding to PSI in order to photosynthesise in low light conditions, emerged. In parallel with the increased light availability, the need for protection mechanisms from excess high light also increased. Therefore, several protection proteins evolved to help the organism to cope with the fluctuating light regime. Moreover, when the freshwater algae evolved to more terrestrial multicellular mosses, excess PSI antennae (LhcA5-9) were lost but some LhcIIs (LhcB9) that could increase the PSI antenna size during low light conditions were acquired. In addition, the protective proteins also acquired more diverse functions. Finally, when higher plants evolved, they acquired very diverse and distinct antenna systems for both PSI and PSII. Moreover, the structural and functional diversity of Lhc superfamily proteins allowed for a very refined and complex fine tuning of light harvesting in response to fluctuating environmental signals. Some understory species have huge antennae and they can cope well with short-term, high light exposure. On the other hand, the species in the canopy layer, where light is abundant, have small antenna sizes and their long-term responses to high light differ from the short-term responses. Moreover, different species have adopted different strategies to thrive in their habitat and have acquired antenna systems and protective proteins based on their needs. However, it is remarkable that basically the same antenna proteins can be adopted to optimize light harvesting in various light environments, as many rather closely related plant species have occupied ecological niches that differ greatly in terms of light quality and quantity. As a future perspective, the organization and function of antenna systems in different organisms across the photosynthetic lineage need to be studied in natural conditions, rather than elucidating the functions of light-harvesting proteins in controlled conditions. This approach may give us a further insight into the strategies that green organisms employ to thrive under diverse environmental conditions and the potential to apply that knowledge to the engineering of plants with enhanced growth.

## Data Availability

Not applicable.
